# Characterizing the Fermentation of Oat Grass (*Avena sativa* L.) in the Rumen: Integrating Degradation Kinetics, Ultrastructural Examination with Scanning Electron Microscopy, Surface Enzymatic Activity, and Microbial Community Analysis

**DOI:** 10.3390/ani15142049

**Published:** 2025-07-11

**Authors:** Liepeng Zhong, Yujun Qiu, Mingrui Zhang, Shanchuan Wei, Shuiling Qiu, Zhiyi Ma, Mingming Gu, Benzhi Wang, Xinyue Zhang, Mingke Gu, Nanqi Shen, Qianfu Gan

**Affiliations:** College of Animal Science, Fujian Agriculture and Forestry University, Fuzhou 350002, Chinashuiling.qiu@aliyun.com (S.Q.); 13763870270@163.com (M.G.); www20210131@163.com (X.Z.); gmk4747@163.com (M.G.);

**Keywords:** *Avena sativa* L., rumen degradation rate, electron microscopy scanning, cellulase, rumen microbiota

## Abstract

The objective of this study is to investigate the degradation characteristics of oat grass in the rumen of Mindong goats and changes in microbial community attached to the grass surface. Four healthy male goats, aged 14 months, with permanent rumen fistula, in eastern Fujian, were selected as experimental animals. The rumen degradation rate of oat grass was measured at 4, 12, 24, 36, 48, and 72 h using the nylon bag method. Surface physical structure changes in oat grass were observed using scanning electron microscopy (SEM), cellulase activity was measured, and bacterial composition was analyzed using high-throughput 16S rRNA gene sequencing technology. The findings of this study indicate that oat grass had effective degradation rates (ED) of 47.94%, 48.69%, 38.41%, and 30.24% for dry matter (DM), crude protein (CP), neutral detergent fiber (NDF), and acidic detergent fiber (ADF), respectively. The SEM was used to investigate the degradation process of oat grass in the rumen. After 24 h, extensive degradation of non-lignified tissue was observed, resulting in the formation of cavities. At 36 h, significant shedding was observed, and by 72 h, only the epidermis and thick-walled tissue, which exhibited resistance to degradation, remained intact. Surface-attached microorganisms produced β-GC, EG, CBH, and NEX enzymes. The activity of these enzymes exhibited a significant increase between 4 and 12 h and showed a positive correlation with the degradation rate of nutrients. However, the extent of correlation varied. *Prevotella* and *Treponema* were identified as key genera involved in the degradation of roughage, with their abundance decreasing over time. Principle Coordinate Analysis (PCOA) revealed no significant differences in the rumen microbial structure across different time points. However, Non-Metric Multidimensional Scaling (NMDS) indicated a discernible diversity order among the samples. According to the Spearman correlation coefficient test, *Ruminococcus*, *Fibrobacter*, and *Saccharoferments* exhibited the closest relationship with nutrient degradation rate and surface enzyme activity, displaying a significant positive correlation. In summary, this study delineates a time-resolved correlative framework linking microbial succession to structural and enzymatic dynamics during oat grass degradation.

## Simple Summary

Understanding how oat grass breaks down in goats’ stomachs helps farmers improve animal diets. We studied this process by placing small oat grass samples into the stomachs of goats through a special opening, and removing them at different times over three days. Using microscopic images and measurements, we discovered the following: (1) Goats digested about half the protein and dry matter in oat grass, but less than a third of tougher fiber parts. (2) The grass structure changed significantly—soft inner tissues broke down within 24 h, leaving only the toughest outer layers after 72 h. (3) Bacteria living on the grass surface produced digestive tools (enzymes) that peaked at specific times, closely linked to how fast nutrients were digested. (4) Different bacteria dominated each digestion stage—early helpers broke down softer tissues, while fiber specialists took over later. By showing exactly how bacteria, enzymes, and grass structure interact hour-by-hour, our findings help explain why oat grass is nutritious for goats despite its toughness. This knowledge can guide farmers in selecting better feed and developing additives to boost digestion, potentially improving animal health and farm productivity.

## 1. Introduction

The rumen serves as the primary functional region within the digestive system of herbivores, boasting a large volume and a complex microbial community comprising bacteria, archaea, fungi, and protozoa. Rumen bacteria occupy a major position in it [1]. These microbial communities play a crucial role in digesting fibrous plant materials and extracting essential nutrients by breaking down complex carbohydrates (e.g., cellulose and hemicellulose) into simple sugars and volatile fatty acids through fermentation processes [2]. This supports up to 70% of the animal’s energy needs [3]. The rumen microbial community is also recognized as a highly promising biochemical resource, capable of producing a variety of cellulose-degrading enzymes, primarily including exo-β-1,4-glucanase, endo-β-1,4-glucanase, and β-glucosidase, showcasing a unique functional adaptability to enhance green biotechnology processes [4].

Microbial attachment is a critical step in degrading plant materials, enabling hydrolytic enzymes secreted by rumen microorganisms to access cellulose substrates. The importance of attachment is underscored by research showing that over 70% of rumen microorganisms adhere to the solid contents of the rumen [5]. This process is initiated by the rumen’s regular peristalsis, which rapidly brings microorganisms into physical contact with newly ingested grass or feed [6], thereby promoting degradation [7]. Therefore, gaining a better understanding of the attached microorganisms and their temporal changes may ultimately aid in the development of new strategies to enhance feed utilization efficiency in ruminants. This multi-modal approach synergistically links microbial identity (16S rRNA sequencing), functional outputs (enzyme activity), and physical degradation patterns (SEM) to provide a mechanistic understanding of how rumen microbiota collaboratively degrade oat grass. Specifically, enzyme activity measurements serve as critical functional indicators that bridge microbial colonization with substrate structural disintegration.

Oat (*Avena sativa* L.), a globally significant forage crop, due to its high nutritional value, elevated protein content, and exceptional palatability, is highly favored by ruminant animals [8]. To date, there have been studies on dynamic degradation and changes in the adherent bacteria colonizing rice straw and alfalfa hay [9], *Artemisia argyi* stalk [10], and wheat straw [11] in the rumen. This study utilized the nylon bag method to assess the rumen degradation characteristics of oat grass. Additionally, high-throughput 16S rRNA gene sequencing was employed to analyze bacterial composition, while scanning electron microscopy was used to observe physical structural changes in oat grass. The study also involved measuring the activity of surface-attached cellulase and exploring their correlations. These efforts aim to provide a theoretical basis and practical guidance for understanding the molecular interactions between rumen microbes and feed substrates, as well as for improving the feed utilization efficiency of ruminants.

## 2. Materials and Methods

### 2.1. Raw Materials

The oat grass was imported from Australia. The whole plant was cut into small segments of approximately 1–2 cm in length, dried at 65 °C, moistened for 2 h, ground, passed through a 40-mesh standard mesh sieve, and stored. The chemical constituents of oat (analyzed value) were DM 89.27%, CP 8.19%, NDF 52.62%, ADF 39.54%, and HC 13.08%.

### 2.2. Animal Feeding

In this study, four 14-month-old healthy Mindong male goats with a body weight of (26.60 ± 2.35 kg) with a permanent rumen fistula were selected. Quantitative feeding was conducted at 09:00 and 17:00 daily, with free access to drinking water. Routine management, immunization, and disinfection of the sheep house were carried out in accordance with epidemic prevention requirements. Before the experiment, there was a 10-day pre-feeding period to balance the rumen population, followed by a 49-day trial period. During the experiment, goat management remained unchanged. The composition and nutritional components of the experimental basal diet are shown in Table 1.

All experimental procedures involving animals were approved by the Animal Care and Use Committee of the School of Animal Science (College of Bee Science), Fujian Agriculture and Forestry University (PZCASFAFU202105, 9 May 2021, Fuzhou, China) and followed the recommendations of the European Commission (1997).

### 2.3. Experimental Design and Sample Collection

Nylon bags with a pore size of 300 mesh and dimensions of 8 cm × 13 cm were selected and numbered. We accurately weighed 4 g of each sample and placed them into prepared nylon bags. Seven time points were established: 4, 8, 12, 24, 36, 48, and 72 h. At each time point, four parallel nylon bags were set up. Four nylon bags were sequentially collected at different time points using the method of ‘synchronous placement, batch removal’. Two of the nylon bags were rinsed with tap water until clarification, and then dried at 65 °C for 48 h to constant weight. After weighing, they were dried and stored for nutrient composition determination and the preparation of scanning electron microscope samples. The other two nylon bags were thoroughly rinsed with distilled water three times to remove rumen fluid and loose microorganisms attached to the surface of the roughage. Sterile gloves were worn to squeeze and remove excess water, which was then frozen in liquid nitrogen and transferred to the laboratory for storage in a −70 °C freezer for subsequent enzyme activity testing and DNA extraction.

### 2.4. Sample Determination and Analysis

#### 2.4.1. Chemical Analysis

The dry matter (DM) and crude protein (CP) contents in the samples were detected using conventional methods [12]. The content of neutral detergent fiber (NDF) and acid detergent fiber (ADF) was determined using the method of Van Soest et al. [13]. Additionally, the hemicellulose content (HC) was calculated as HC = NDF − ADF.

#### 2.4.2. Ruminal Degradability Analysis

The degradability, degradation parameters, and effective degradability of DM, CP, NDF, and ADF in the rumen of goats were calculated.

For the degradation rate of oat grass at different time points, the formula is as follows:

A (%) = 100 × (B − C)/B × 100%(1)

In the formula, A represents the degradation rate of the sample to be tested (%), B represents the nutritional content in the sample (%), and C represents the residual content in the nylon bag sample (%).

According to the method explained by Ørskov et al. [14], the rumen degradation kinetic parameters and effective degradation rate were estimated. The formula is as follows:

Rates of rumen degradation: P = a + b × (1 − e^−ct^) (2)

Effective degradation rate: ED (%) = a + [bc/(c + k)](3)

In the formula, “P” represents the rumen degradation rate of the sample at time t; “a” represents the rapid degradation rate of the sample to be tested (%); “b” represents the slow degradation rate (%); “c” represents the degradation rate of the slow degradation part (%/h); “ED” represents the effective degradation rate of the sample to be tested (%); “k” represents rumen outflow rate %/h; and “k” represents 0.235%/h in this experiment [15].

#### 2.4.3. Scanning Electron Microscopy (SEM)

The samples digested by the rumen in the nylon bag were taken out for SEM analysis. First, the samples were fixed in a 2.5% glutaraldehyde solution for 24 h. The next day, the glutaraldehyde fixative was poured out, and the fragments were rinsed three times with phosphate buffer, dehydrated with ethanol at all levels, treated with a 1:1 mixture of isoamyl acetate and ethanol for 30 min, and then treated with pure isoamyl acetate for 1 h. After drying at the critical point, the sample table was sprayed with gold and the samples were observed and photographed using a JEOL JSM-6390A (purchased from JEOL Ltd., Tokyo, Japan) scanning electron microscope.

#### 2.4.4. Cellulase Activity Assay

We collected samples and analyzed changes in enzyme activity during the rumen degradation process. We selected 4 enzymes related to cellulose degradation: β-Glucosidase (BG enzyme), Neutral xylanase (NEX enzyme), Endo-β-1,4-glucanase (C1 enzyme), and Exo-β-1,4-glucanase (Cx enzyme). All steps of enzyme activity level determination were carried out using a reagent kit (purchased from Quanzhou Ruixin Biotechnology Co., Ltd., Quanzhou, China).

Specifically, the measurement procedures for BG and NEX enzymes were as follows: Approximately 0.1 g of sample was weighed, mixed with 1 mL of extraction solution, and homogenized in an ice bath. The homogenate was centrifuged (4 °C, 12,000 rpm, 10 min), and the supernatant was retained. Enzyme activity was then determined using a commercial reagent kit (Ruixin Biotechnology, Quanzhou, China) and a SpectraMax 17 microplate reader (Molecular Devices, New York, NY, USA).

The steps for measuring the C1 enzyme and Cx enzyme are as follows: Weigh approximately 0.2 g of sample, add 1 mL of pre-cooled 95% ethanol ice bath homogenate, place at 4 °C for 10 min, then centrifuge (4 °C, 12,000 rpm, 5 min). Abandon the supernatant and retain the sediment. Add pre-cooled 80% ethanol to the precipitate, mix well, place at 4 °C, and then centrifuge (4 °C, 12,000 rpm, 5 min). Discard the supernatant and retain the sediment. Add 1 mL of pre-cooled extraction solution to the precipitate, vortex-mix well, place at 4 °C for 10 min, and centrifuge (4 °C, 12,000 rpm, 10 min). Retain the supernatant and use a reagent kit and SpectraMax 17 microplate reader (MD, New York, NY, USA) to determine.

#### 2.4.5. Rumen Microbiome Analysis Methods

Using the MagPure Soil DNA LQ Kit (Magen, Shanghai, China), genomic DNA was extracted from nylon bags containing oat grass. To analyze microbial diversity, the V3-V4 variable region of the 16S rRNA gene was amplified using universal primers 343F (5′-TACGGRAGGCAG-3′) and 798R (5′-AGGGTATCTAATCCT-3′). The amplified products were purified and sequenced on the NovaSeq 6000 platform (Illumina, Inc., San Diego, CA, USA). The construction, sequencing, and data analysis of the database were carried out by OE Biotechnology Co., Ltd. (Shanghai, China). The raw data was in FASTQ format. After downloading the data, we first used cutadapt software 4.5 [16] to cut out the primer sequence for the raw data sequence. Then, we used DADA2 [17] to perform quality filtering, noise reduction, splicing, and quality control analysis on the qualified dual-end raw data from the previous step according to the default parameters of QIIME 2 (2020.11) [18]. We obtained representative sequences and ASV abundance tables. The Silva 16S rRNA database (v. 138) was used for comparison, and the species comparison annotations were analyzed using the default parameters of the q2-feature-classifier software 2021.8.0. Microbial community diversity was analyzed by QIIME 2. Alpha diversity was evaluated by Chao1, ACE, and Shannon and Simpson indexes. The beta diversity was calculated by weighted and unweighted Unifrac distance matrices, and evaluated by principal component analysis (PCA) cluster analysis. LEfSe 1.1.2 was used for species abundance analysis of variance, and PICRUSt2 [19] was used for functional abundance prediction. Functional predictions via PICRUSt2 were inferred from 16S rRNA gene profiles and represent hypotheses about potential metabolic capabilities, not direct measurements of gene expression or enzyme activities.

### 2.5. Statistical Analysis Method

This study used Excel 2020 software to organize the initial data, SPSS 26.0 for one-way ANOVA, and Duncan’s method for multiple comparisons. The results are expressed as mean ± standard deviation, with *p* < 0.05 indicating significant differences. We further evaluated degradation kinetics using the nonlinear model in SPSS 26.0 software, and conducted post hoc analysis using Duncan’s multiple comparison test.

Various R packages (v4.3.0, ggplot2, linkET) contribute to PCA, mapping, Unifrac distance matrix results, and graphical analysis of correlation network heat maps. The Spearman rank correlation coefficient was used to test the relationship between variables, and the correlation between the main bacterial genera and nutrient degradation was proven using a graph-based testing method.

## 3. Results

### 3.1. Ruminal Degradability

According to Table 2, DM, CP, NDF, and ADF degraded slowly at 4~8 h (*p* > 0.05) and rapidly degraded within 12~24 h (*p* < 0.05); they had different degradation rates within 36 h, but all tended to stabilize within 48~72 h (*p* > 0.05). The whole presents a slow–fast–slow degradation process. Among the degradation parameters, CP has the highest rapid degradation rate (“a”) at 16.31%. The slow degradation (“b”) of DM is significantly higher than that of CP, NDF, and ADF. Additionally, the effective degradation rate (“ED”) of CP is the highest at 48.69%.

### 3.2. Scanning Electron Microscopy (SEM) Observations

**Figure 1:**
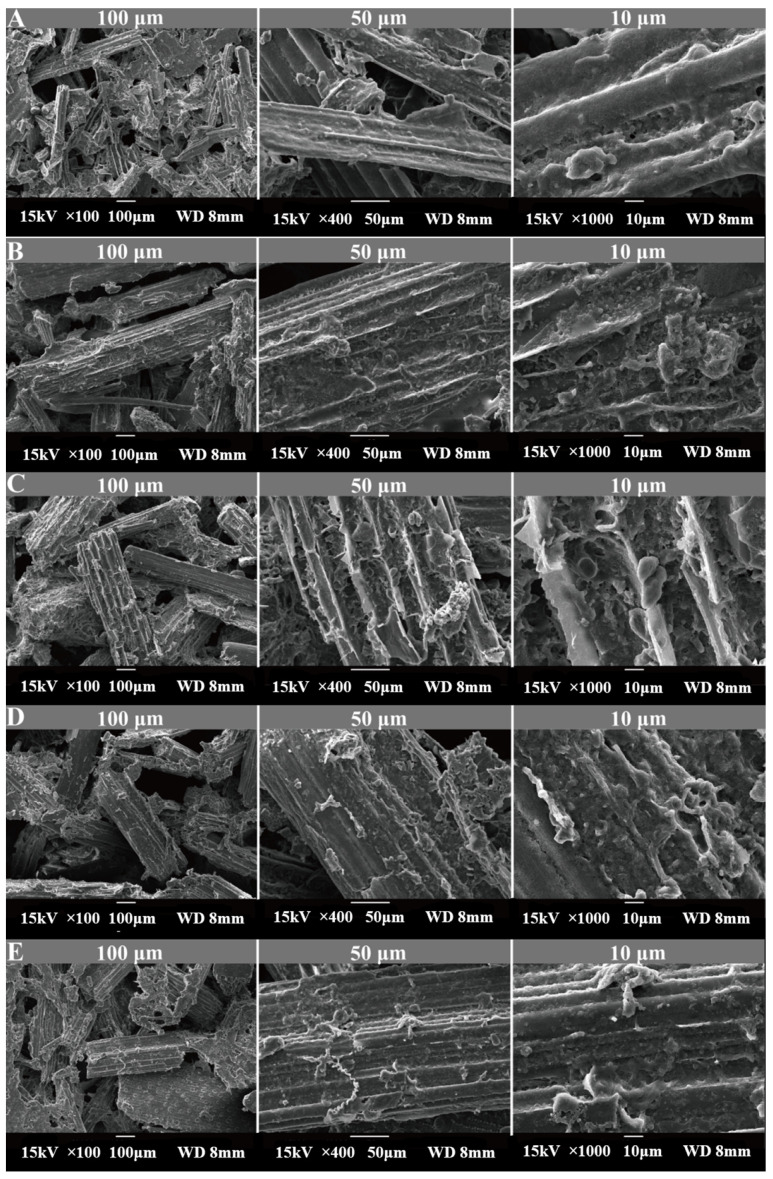
Changes in the structure and tissue of oat grass under SEM: (**A**) 4 h; (**B**) 12 h; (**C**) 24 h; (**D**) 36 h; (**E**) 72 h. At each time point, pictures of oat structure under 100 μm, 50 μm, and 10 μm microscope scales are displayed.

Figure 1A–E illustrates the dynamic digestion process of oat grass in the rumen of goats as observed under SEM. Oat grass comprises the following components: epidermis, sclerenchyma, vascular bundle (bundle sheath, xylem, phloem), and parenchyma. Within 4~12 h, the degradation of parenchyma tissue becomes noticeable, accompanied by the gradual dissolution of peripheral vascular bundles (Figure 1A,B). By 24 h, a distinct cross-section can be observed with degradation occurring in the phloem and xylem, resulting in the formation of cavities at the degradation site (Figure 1C). The vascular bundle structure at 36 h demonstrates significant shedding following the degradation of the surrounding parenchyma (Figure 1D). At 72 h, only the hard-to-degrade epidermis and thick-walled tissue remain intact (Figure 1E).

Quantitative analysis of SEM images revealed progressive structural disintegration. Cell wall thickness decreased by 38.2% in the parenchyma (4 h: 2.15 ± 0.34 μm→24 h: 1.33 ± 0.21 μm, *p* < 0.01). Pore area in vascular bundles increased 5.7-fold from 4 h to 36 h (4 h: 8.3 ± 2.1 μm^2^→36 h: 47.5 ± 9.4 μm^2^, *p* < 0.001). Epidermal lignin coverage (72 h) reached 72.4 ± 6.3%, explaining residual recalcitrance (vs. 4 h: 22.1 ± 3.8%, *p* < 0.001).

### 3.3. Cellulase Activity Analysis

**Figure 2:**
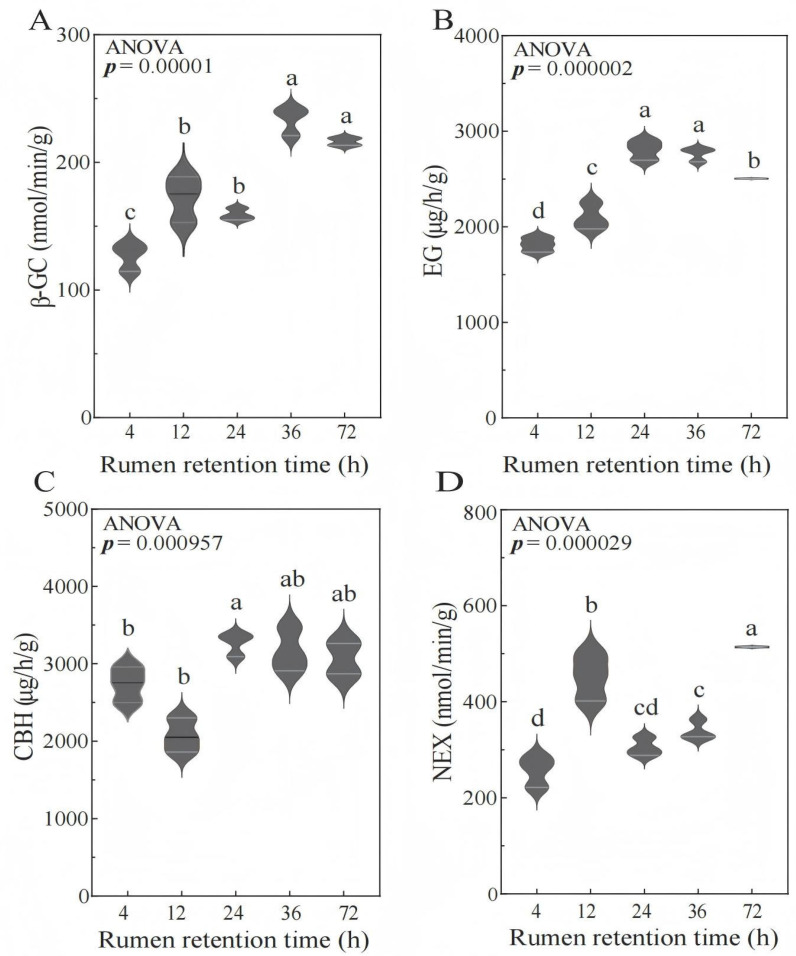
Cellulase activity attached to the surface of oat during rumen degradation: (**A**) β-Glucosidase; (**B**) endo-1,4-β-D-glucanohydrolase; (**C**) exo-1,4-β-D-glucanase; (**D**) Neutral xylanase. Different lowercase letters in the same row indicate significant differences (*p* < 0.05).

As shown in Table 3 and Figure 2, the β-GC enzyme activity significantly increased from 4 to 24 h (*p* < 0.05) and tended to stabilize from 36 to 72 h. The activity of EG and CBH enzymes significantly increased from 4 to 12 h (*p* < 0.05), followed by a gradual plateau from 24 to 36 h. In addition, the activity of the EG enzyme significantly decreased at 72 h (*p* < 0.05). The NEX enzyme activity increased from 4 to 12 h (*p* < 0.05), then decreased, and significantly increased at 72 h (*p* < 0.05), showing a curve pattern of first increasing, then decreasing, and then increasing.

These temporal enzyme activity profiles reflect stage-specific microbial colonization and fiber deconstruction processes.

Early colonization phase (4–12 h): The rapid increase in EG and CBH activities (*p* < 0.05) coincides with initial bacterial attachment to accessible cellulose microfibrils (Figure 1A,B), facilitating hemicellulose loosening and amorphous cellulose hydrolysis.

Peak degradation phase (12–36 h): Sustained high β-GC and EG levels (*p* < 0.05) correlate with maximal cavitation in non-lignified tissues (Figure 1C,D), driven by the synergistic action of Ruminococcus and Fibrobacter (as will be detailed in Section 3.4) which secrete exo-acting enzymes targeting crystalline cellulose.

Residual fiber phase (48–72 h): The secondary rise in NEX activity (*p* < 0.05) corresponds to Saccharofermentans enrichment (as will be detailed in Section 3.4) and reflects microbial adaptation to recalcitrant hemicellulose–lignin complexes in epidermal residues (Figure 1E).

### 3.4. Rumen Microbiome Analysis

This study acquired a total of 1,580,734 labels from 20 samples. After control and filtration, 918,017 clean labels were obtained. Subsequently, further analysis was conducted to examine the diversity of rumen microbiota attached to oat surfaces at different time points (Table 4). For all samples, the average coverage rate exceeded 99%, enabling further analysis of microbial community changes. The dynamic changes in alpha diversity revealed no statistically significant differences in the Chao1, ACE, Shannon, and Simpson index of the rumen microbiota at different time points (*p* > 0.05).

**Figure 3:**
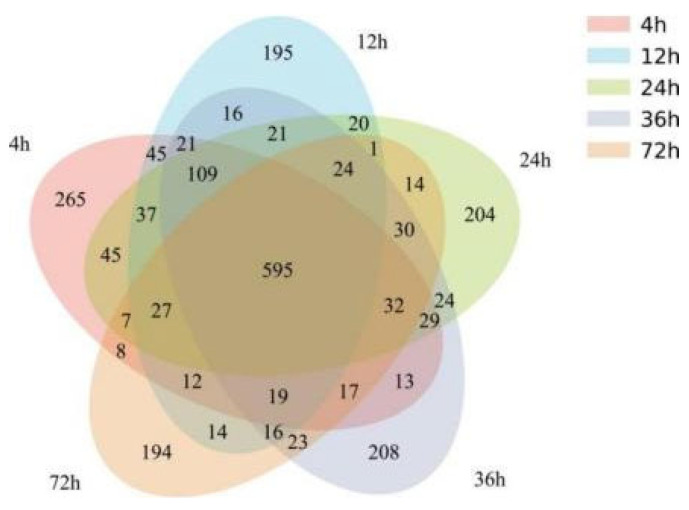
OTU Venn diagram analysis.

According to Venn analysis (Figure 3), there were a total of 595 OTUs in the rumen culture process, with 195, 204, 208, 194, and 265 unique OTUs at 4, 12, 24, 36, and 72 h, respectively.

**Figure 4:**
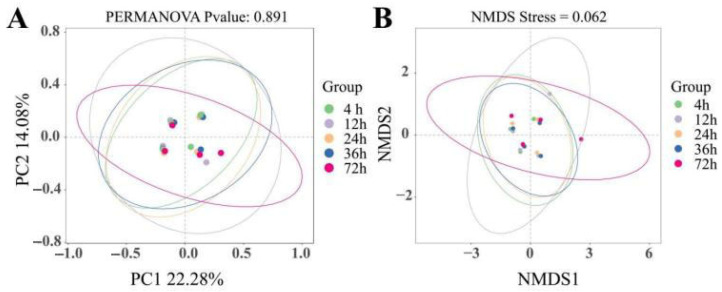
Principal coordinate analysis (PCoA) and non-metric multidimensional analysis (NMDS) of rumen microbial community structure at different time points for oat based on unweighted unifrac distance: (**A**) PCoA; (**B**) NMDS.

**Figure 5:**
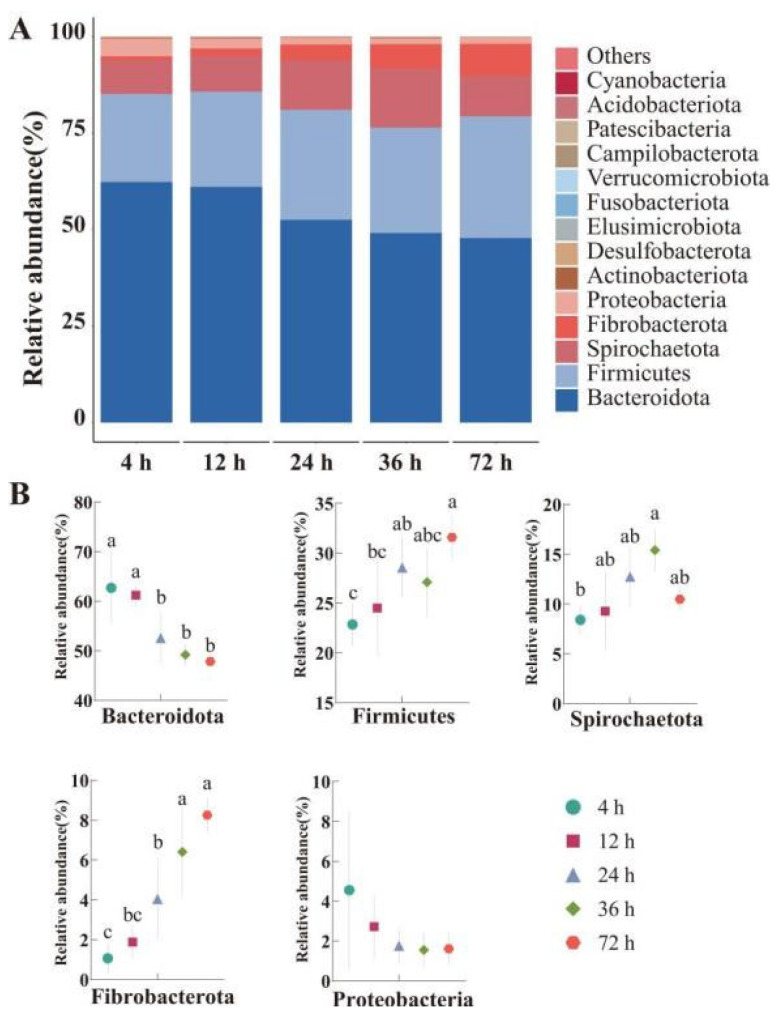
Relative abundance of phylum-level microorganisms (%): (**A**) Phylum-level taxonomic relative abundance variation; (**B**) Changes in relative abundance at the phylum level. Different lowercase letters in the same row indicate significant differences (*p* < 0.05).

While alpha diversity indices (Chao1, ACE, Shannon; Table 4) and beta diversity ordination (PCoA/NMDS; Figure 4) showed no significant temporal shifts (*p* > 0.05), phylum-level and genus-level compositional changes revealed functional adaptation to substrate degradation stages. At the phylum level (Figure 5A,B), it can be seen that Bacteroidota, Firmicutes, Spirochaetota, Fibrobacterota, and Proteobacteria were the top five dominant bacteria, with relative abundance higher than 1%, accounting for 99.62% of the total bacterial phylum. Among them, the Bacteroidota at 4 h and 12 h was significantly higher than at other time points (*p* < 0.05). As time went on, the abundance of Bacteroidota decreased (*p* < 0.05), while the abundance of Firmicutes increased (*p* < 0.05). At 72 h, the abundance of Firmicutes was significantly higher than at 4 and 12 h (*p* < 0.05). The abundance of Spirochaeota reached its peak at 36 h (*p* < 0.05). The abundance of Fibrobacterota reached its peak at 72 h, with no significant difference from 36 h (*p* > 0.05), but was significantly higher than at 4, 12, and 24 h (*p* < 0.05). There was no significant difference in Proteobacteria among different time points (*p* > 0.05).

**Figure 6:**
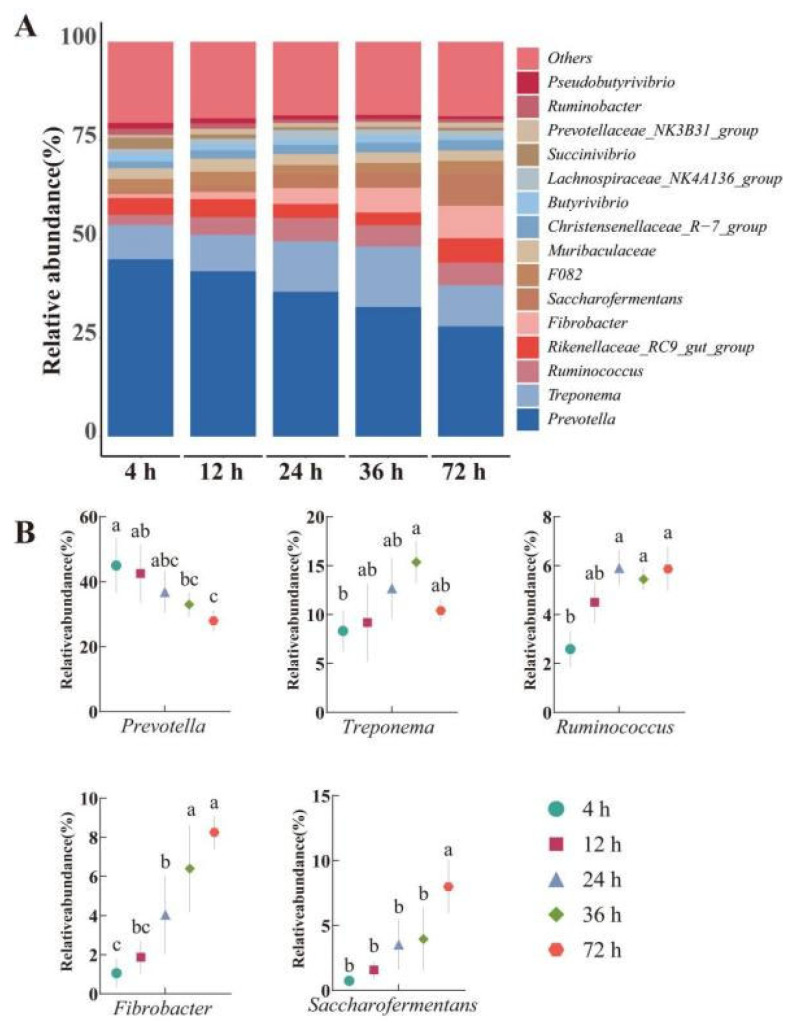
Relative abundance of genus-level microorganisms (%): (**A**) Genus-level taxonomic relative abundance variation; (**B**) Changes in relative abundance at the genus level. Different lowercase letters in the same row indicate significant differences (*p* < 0.05).

At the genus level (Figure 6A,B), we selected bacterial genera with relative abundance higher than 1% for analysis. At each time point, Prevotella was the genus with the highest relative abundance, accounting for 27.95% to 44.94%. Additionally, the relative abundance of *Prevotella* at 4 h was significantly higher than that at 72 h (*p* < 0.05). The relative abundance of Treponema reached its peak at 36 h and was significantly higher than at 4 h (*p* < 0.05). In addition, the relative abundance of Ruminococcus and Fibrobacter at 24, 36, and 72 h was significantly higher than that at 4 h (*p* < 0.05). The relative abundance of Saccharoferments reached its highest at 72 h and was significantly higher than other groups (*p* < 0.05). There was no significant difference in the relative abundance of the other genera at different time points (*p* > 0.05).

**Figure 7:**
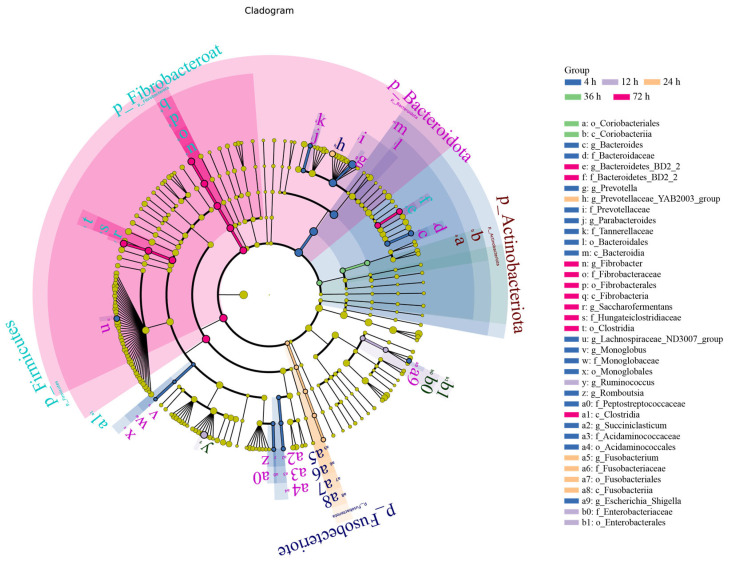
LEfSe analysis branch graph at different time points.

LEfSe (Figure 7) with FDR correction (q < 0.05) confirmed the temporal specialization of fiber-degrading taxa: Early phase (4 h): *Prevotella* (LDA = 4.2, q = 0.008) dominated hemicellulose breakdown, aligning with initial NEX surge (Table 3). Mid phase (24–36 h): *Ruminococcus* (24 h: LDA = 4.1, q = 0.022) and *Fibrobacter* (36 h: LDA = 4.8, q = 0.003) correlated with crystalline cellulose degradation (β-GC/EG peaks, Table 3) and vascular bundle shedding (Figure 1D). Late phase (72 h): Saccharofermentans (LDA = 4.3, q = 0.015) increased as epidermal lignin exposure intensified (Figure 1E), coinciding with secondary NEX rise. Non-fiber-related taxa (e.g., Escherichia_Shigella, q = 0.21; Fusobacterium, q = 0.17) were excluded from analysis as non-significant after FDR adjustment.

### 3.5. The Correlation Between Nutrient Degradation Rate, Cellulase Activity, and Rumen Bacteria

**Figure 8:**
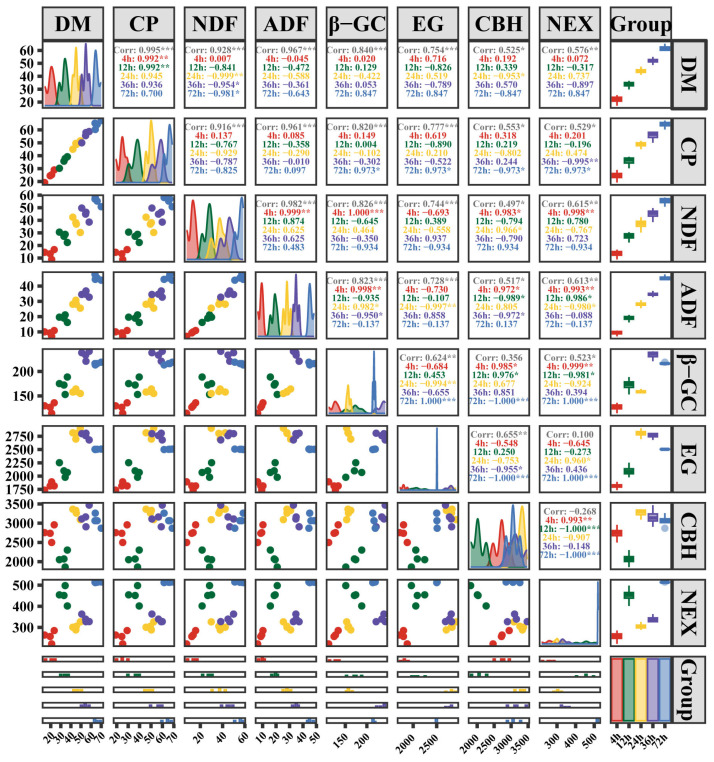
Analysis of the composite correlation plot of nutrient degradation rate and enzyme activity. Note: Arranged from right to left, these are box plots, correlation analysis data, density plots, and scatter plots, with histograms at the bottom. These visual representations depict the distribution and correlation of nutrient degradation rates and enzyme activity at 4, 12, 24, 36, and 72 h from various perspectives. The “corr” indicate the correlation coefficient (range from −1 to 1); “*” represents *p* < 0.05; “**” represents *p* < 0.01; and “***” represents *p* < 0.001.

To further analyze the correlation between nutrient degradation rate, cellulase activity, and rumen bacteria, this study first conducted a correlation analysis between the degradation rate of nutrients in the rumen and surface enzyme activity (Figure 8). Overall (4–72 h), the results showed a positive correlation between nutrients and degradation rates, though the strength of this correlation varied. Specifically, four nutrients (DM, CP, NDF, ADF) demonstrated a highly significant positive correlation with β-GC and EG (r > 0.82, *p* < 0.001) and a significant correlation with CBH (r > 0.50, *p* < 0.05). For NEX, there was a highly significant correlation with DM, NDF, and ADF (r > 0.58, *p* < 0.01) and a significant correlation with CP (r = 0.529, *p* < 0.05).

Across various time frames, the relationship between surface enzyme activity and degradation rate exhibited dynamic shifts at five distinct time points, reflecting the intricate nature of digestion in the rumen. At 4 h, the degradation rates of DM and CP were positively correlated with EG, but there was no significant difference (r > 0.62, *p* > 0.05). The degradation rates of NDF and ADF were both correlated with EG, β-GC, and CBH, and NEX showed a significant positive correlation (r > 0.97, *p* < 0.05). At 12 h, ADF was negatively correlated with CBH (r = −0.989, *p* < 0.05), and positively correlated with NEX (r = 0.986, *p* < 0.05). At 24 h, CBH and DM showed a negative correlation (r = −0.953, *p* < 0.05), while NDF showed a positive correlation (r = 0.966, *p* < 0.05). ADF was negatively correlated with EG and NEX (r = −0.997, r = −0.98, *p* < 0.05). At 36 h, the degradation of ADF was significantly negatively correlated with β-GC and CBH (r = −0.95, r = −0.972, *p* < 0.05), while CP was negatively correlated with NEX (r = −0.995, *p* < 0.01). At 72 h, the degradation rate of CP was significantly positively correlated β-GC, EG, and NEX, and negatively correlated with CBH (|r| > 0.97, *p* < 0.05).

**Figure 9:**
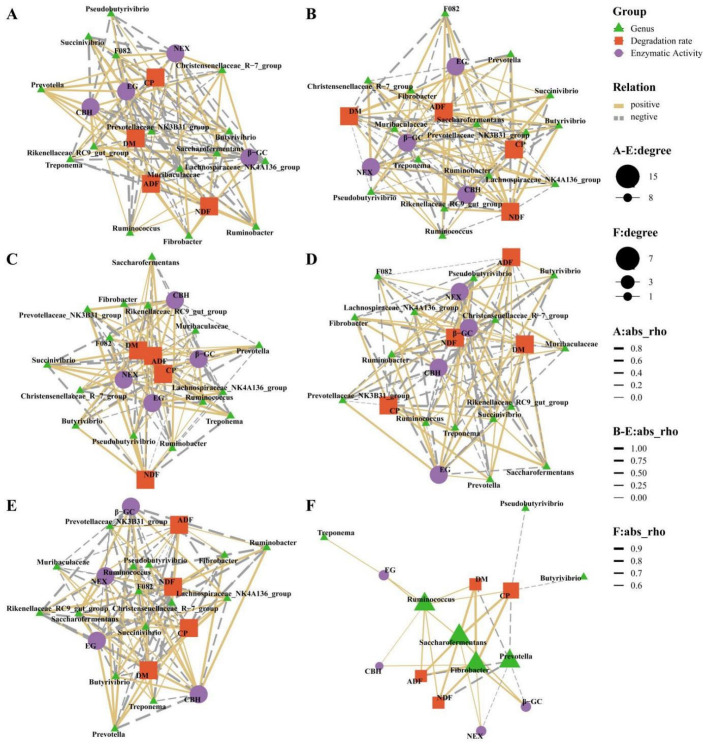
The correlation between nutrient degradation rate, cellulase activity, and rumen bacteria. Blue represents negative correlation, while pink represents positive correlation: (**A**) 4 h; (**B**) 12 h; (**C**) 24 h; (**D**) 36 h; (**E**) 72 h; (**F**) 4–72 h.

Later, Spearman’s correlation coefficient was used to test the relationship between the relative abundance of the top 5 phylum and top 15 genus bacteria and nutrient degradation rate and enzyme activity (Figure 9). To visualize the dynamic interrelationships among the three more clearly, only the significance levels are marked in the graph. Research shows that during the whole process of 4–72 h, the degradation rates of four nutrients were negatively correlated with *Bacteroidota*, *Prevotella*, and *Treponema* (|r| > 0.5, *p* < 0.05), and significantly positively correlated with Firmicutes, Fibrobacterota, Ruminococcus, Fibrobacter, and Saccharofermentans (r > 0.5, *p* < 0.05). Both DM and CP were negatively correlated with Ruminobacter and Pseudobutyrivibrio (|r| > 0.5, *p* < 0.05), and CP was also negatively correlated with Butyrivibrio (r = −0.452, *p* = 0.045). NDF was negatively correlated with Christensenellaceae_R-7_group and Succinivibrio (|r| > 0.4, *p* < 0.05). ADF was negatively correlated with Pseudobutyrivibrio (r = −0.501, *p* = 0.024). Regarding the relationship between enzyme activity and microorganisms, a positive correlation (r > 0.5, *p* < 0.05) was observed between the four groups of enzyme activities and Fibrobacterota. β-GC, EG, and CBH all showed a negative correlation with Bacteroidota (|r| > 0.5, *p* < 0.05). Additionally, β-GC and EG demonstrated a positive correlation with Ruminococcus and Saccharofermentans, while also exhibiting a negative correlation with Proteobacteria and Prevotella (|r| > 0.5, *p* < 0.05). In contrast, EG was positively correlated with Firmicutes, Spirochaetota, and Treponema (r > 0.5, *p* < 0.05); NEX was positively correlated with Firmicutes and Saccharofermentans (r > 0.5, *p* < 0.05). Moreover, at 72 h, the association between β-GC/EG/NEX on one hand, and Bacteroidota and Prevotella on the other, was reciprocal (|r| > 0.5, *p* < 0.05).

Focusing on causally plausible relationships, Fibrobacter abundance strongly correlated with CBH activity (r = 0.82, *p* < 0.001), aligning with its cellulosome machinery containing exoglucanase modules. Ruminococcus was linked to β-GC activity (r = 0.79, *p* < 0.01), consistent with its β-glucosidase secretion for cellobiose hydrolysis. Both genera drove NDF degradation (r > 0.80, *p* < 0.001) via crystalline cellulose breakdown (SEM, Figure 1D). Other statistically significant correlations (e.g., Pseudobutyrivibrio vs. DM) lacked clear mechanistic support and were omitted. This suggests that the correlations varied across different time points and the entire duration, reflecting the dynamic degradation processes within the rumen.

### 3.6. Functional Prediction

**Figure 10:**
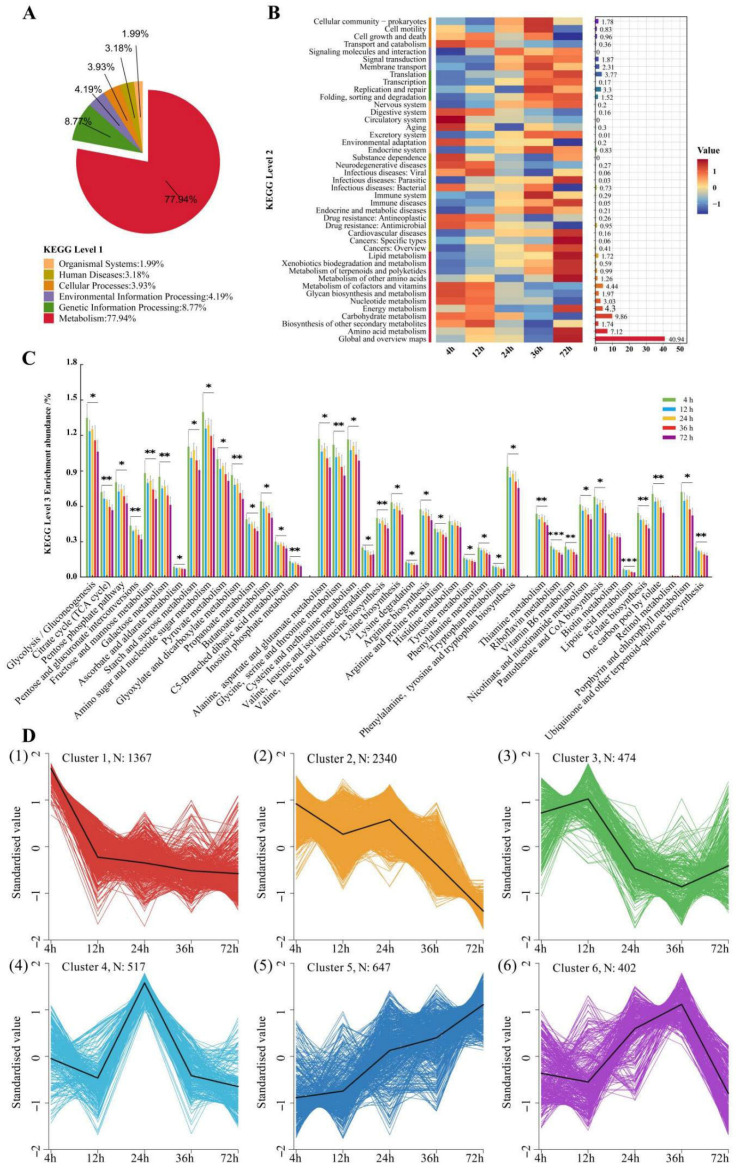
The function of the rumen microbiota in experimental goat samples was predicted using PICRUSt2 software: (**A**) KEGG Top-level pathways; (**B**) KEGG Level 2 pathways; (**C**) KEGG Level 3 pathways; (**D**) KO-based K-means clustering heatmap. * indicates *p* < 0.05, ** indicates *p* < 0.01, *** indicates *p* < 0.001. For the classification cluster, *n* is the number, the ordinate indicates the normalized material content, and the best number of clusters is calculated according to the Calinski–Harabasz algorithm.

As a note of caution, PICRUSt2-based predictions reflect computationally inferred functional potentials rather than experimentally confirmed biochemical activities. The function of the rumen microbiota in experimental goat samples was predicted using PICRUSt2 software v.2.5.3 (Figure 10). The hierarchical classification statistics of pathways revealed that, at level 1, the functional genes of each group were predominantly enriched in the metabolism (77.94%) (Figure 10A). At the KEGG L2 level, 43 metabolic pathways were displayed (Figure 10B). Among them, the top five metabolic pathways were global and overview maps (40.94%), carbohydrate metabolism (9.86%), amino acid metabolism (7.12%), metabolism of coenzymes and vitamins (4.44%), and energy metabolism (4.3%). Among them, it was found that the carbohydrate metabolism pathway was significantly higher at 4–24 h than at 72 h (*p* < 0.05), and the amino acid metabolism at 36 h was significantly lower than at 72 h (*p* < 0.05). The maximum metabolism content of cofactors and vitamins at 4 h was significantly higher than that at 36–72 h (*p* < 0.05) (Figure 10B). Therefore, this experiment further searched for the KEEG L3 pathway among these three metabolic pathways and found that there were 15, 14, and 12 pathways, respectively (Figure 10C). Among them, the top three pathways in carbohydrate metabolism are amino sugar and nucleotide sugar metabolism (1.25%), glycine/gluconeogenesis (1.21%), and starch and sucrose metabolism (1.02%). In amino acid metabolism, the top three pathways are cysteine and methionine metabolism (1.07%), alanine, aspartate, and glutamate metabolism (1.05%), and glycine, serine, and threonine metabolism (0.99%). In the metabolism of cofactors and vitamins, the top three pathways are one carbon pool by folate (0.62%), porphyrin and chlorophyll metabolism (0.62%), and pantothenate and CoA biosynthesis (0.61%). Within these metabolic pathways, the relative abundance peaked at 4 h, then gradually decreased, and was significantly higher than that at 72 h (*p* < 0.05).

For further analysis, 5747 KOs were subjected to the Kruskal–Wallis test and Calinski–Harabasz algorithm. When divided into six classes, classes 1~3 showed a downward trend, classes 4 and 6 showed first a rise and then a decrease, which peaked at 24 and 36 h, respectively, and 647 in class 5 showed an upward trend.

### 3.7. Correlation Analysis Between KEGG and Nutrition, Enzyme Activity, and Bacteria

**Figure 11:**
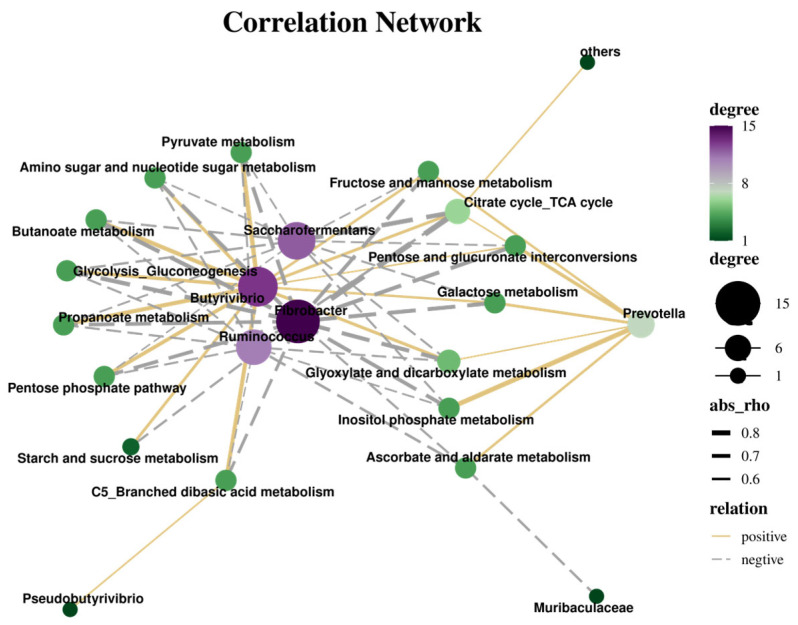
Network analysis of correlation between carbohydrate metabolism pathways and rumen flora. Note: The size and color depth of the circle (green to purple) indicate the extent of the associated object in the legend to the right. Purple and green nodes indicate centrality. Lines connected to the nodes indicate a significant correlation between the species, and the thickness of the lines indicates the strength of the correlation (|r| > 0.5, *p* < 0.05). Yellow and silver lines indicate positive and negative correlations, respectively.

The correlation analysis of the interaction between carbohydrate metabolic pathways and the rumen bacterial flora is shown in Figure 11. In oat grass, 22 pathways were positively correlated and 39 were negatively correlated (r > 0.5, *p* < 0.05). And 13 of the carbohydrate metabolism pathways involved (r > 0.5, *p* < 0.05) were Pyruvate metabolism, propionate metabolism, C5 branching diacid metabolism, pentose phosphate pathway, glycolysis/glyconin (Glycolysis/Gluconeogenesis), metabolism of glyoxylate and dicarboxylate (Glyoxylate and dicarboxylate metabolism), citric acid cycle (TCA cycle) (Citrate cycle (TCA cycle)), starch and sucrose metabolism, fructose and mannose metabolism (Fructose and mannose metabolism), and the interconversion pathways of galactose metabolism, pentose, and glucuronides. *Prevotella* was positively associated with seven carbohydrate metabolism pathways (r > 0.5, *p* < 0.05), phosphoinositol metabolism, galactose metabolism, ascorbate and aldehydes metabolism, fructose and mannose metabolism, interconversion of pentose and glucurononate, citric acid cycle (TCA cycle), glyoxylate and dicarboxylate metabolism, respectively, and in addition, pseudobutylvibrio (Pseudobutyrivibrio) was positively correlated with the C5 clade diacid metabolism pathway (r = 0.53, *p* < 0.05).

## 4. Discussion

The rumen degradation rate serves as a crucial indicator reflecting the physiological activities of rumen microbes [20] and their relationship to feed intake in ruminants [21]. The rapid degradation of dry matter (DM) partly reflects the biodegradable non-structural carbohydrate content in the feed, while the effective degradability (ED) indicates the digestibility expected in vivo [22]. Generally, higher DM degradation rates correlate with increased dry matter intake in ruminants [23]. In this study, the degradation rate of oat grass stabilized from 48 to 72 h, indicating that ruminal microorganisms degraded most digestible dry matter within 48 h of fermentation. The 72 h degradation and ED values were 65.76% and 47.94%, respectively, which are lower than those reported for oatgrass (70.80% and 50.50%) [24], but higher than values for Chinese Rye Grass (60.81% and 46.53%), Barley Grass (60.33% and 40.89%), and Naked Oat Straw (58.63% and 44.90%) in a study [25] by Yulin Ma et al., with (60.81%, 46.53%), Macrograss Barley Grass (60.33%, 40.89%), and Naked Oat Straw (58.63%, 44.90%). This indicates that the digestion of oat grass in the rumen may be influenced by many factors, such as the rumen environment, grass origin, variety. and animal species. However, it still has a high forage value compared to other grasses. Dietary CP disappearance is related to raw material characteristics, such as anti-trophic factors and cell wall barrier effects. The study by Ma et al. [25] showed that the effective degradation rate of CP had the highest correlation with its 24 h rumen degradation rate. CP’s 24 h degradation peak reflects its high solubility, but is constrained by anti-nutritional barriers: Phenolic compounds in oat grass may bind proteins, limiting microbial access to chloroplast proteins (SEM shows intact chloroplasts at 12 h, Figure 1B). Rapid ammonia assimilation by Prevotella reduces proteolysis efficiency post-24 h. NDF and ADF are important parameters to measure the degree of feed structural carbohydrate utilization, and their value size can reflect the digestion of feed fiber components in the rumen. In this study, the degradation rates of NDF and ADF at 72 h were 55.24% and 45.32%, respectively, which is lower than those of DM and CP at the same time (65.76% and 64.16%), probably due to the complex structure of fiber and the high acid-washing fiber content; acid-washing fiber contains cellulose and lignin, and lignin cannot be completely absorbed. The closely linked combination of lignin, cellulose, and hemicellulose reduces microbial utilization efficiency, leading to prolonged rumen retention time and low degradation rates [26]. Additionally, some studies suggest that dry matter degradation rate is positively correlated with CP content, while showing negative correlations with NDF and ADF content, although the correlation strengths vary [27]. Our results demonstrate that temporal correlations generally align with this pattern.

After introducing the sample into the rumen, numerous ruminal microorganisms rapidly colonize the cell wall surface and secrete exoenzymes. Cellulase—a complex enzyme system that hydrolyzes cellulose to glucose—encompasses three functional classes based on catalytic activity: endoglucanase (EC 3.2.1.4), exoglucanase (EC 3.2.1.91), and β-glucosidase (EC 3.2.1.21). In this study, endoglucanase (EG) and exoglucanase (CBH) activities exhibited an initial increase followed by a decline, peaking at 24 h. β-glucosidase (β-GC) displayed a biphasic pattern (“M-shaped” curve). Xylanases, which hydrolyze the dominant hemicellulose component xylan [28], demonstrated ruminal hemicellulose disappearance. Notably, neutral xylanase (NEX) activity showed a distinct increasing trend compared to other enzymes. These enzymatic dynamics were temporally aligned with the structural degradation observed via scanning electron microscopy.

Additionally, after 72 h of ruminal degradation, the sclerenchyma tissue beneath the epidermis remained intact with minimal degradation. This resilience primarily stems from the thickened, highly lignified cell walls developed during tissue maturation, which severely restrict microbial activity even when microorganisms penetrate the cell lumen. These findings elucidate the degradation constraints of oat grass in the rumen, suggesting that targeted enzyme supplementation could enhance cellulose breakdown.

The plant cell wall matrix—comprising recalcitrant cellulose, hemicellulose, and lignin complexes—poses the greatest degradation challenge in ruminants. Surface barriers like the cuticular wax layer further impede rapid microbial and enzymatic attack. Rumen microorganisms primarily exploit damaged surfaces and stomata to initiate degradation. Consequently, inner cell layers degrade later due to compact cell packing and wall overlapping. Crucially, cell wall rupture exposes cellular contents, which are essential for nutrient release and feed additive efficacy [29].

Ruminal carbohydrate degradation involves synergistic microbial consortia, with temporal colonization shifts reflecting substrate modification and functional adaptation. Jin et al. [11] documented biphasic (fast-slow) degradation in wheat straw, while our study revealed triphasic slow–fast–slow kinetics in oat grass. This pattern aligns with dried straw and alfalfa hay [9], but diverges markedly from fresh perennial ryegrass [30]. Critically, the sequential substrate degradation observed via SEM directly mirrors enzymatic and microbial dynamics. Early non-lignified tissue degradation (4–12 h, Figure 1B) was driven by Prevotella-secreted NEX enzymes (r = 0.86 vs. hemicellulose degradation, *p* < 0.01), solubilizing hemicellulose to expose cellulose microfibrils. Vascular bundle cavitation (24–36 h, Figure 1C,D) coincided with Fibrobacter proliferation (3.8→9.1%, Figure 6B) and CBH/β-GC peaks (Table 3), whose cellulosomes hydrolyze crystalline cellulose. Epidermal persistence (72 h, Figure 1E) selected for Saccharofermentans (LDA = 4.3, Figure 7) via lignin-modifying laccases that reactivate NEX (r = 0.79, *p* < 0.01) for residual xylan cleavage. This tripartite synergy, where SEM structures dictate microbial functional succession and enzyme activities serve as real-time biochemical reporters, establishes a novel mechanistic framework for fiber degradation stages. This experiment studied the composition of rumen microbial species at different time points during the entire digestion process of oat grass and identified Bacteroidetes, Firmicutes, Spirochaetes, Fibrobacter, and Proteobacteria as the dominant phyla, with a total bacterial count of up to 99.70%. The presence and function of these phyla in the rumen play a crucial role in the digestion and utilization of plant cellulose by ruminants. Bacteroidetes mainly participate in the degradation of carbohydrates, secreting various enzymes to break down cellulose, thereby improving the utilization rate of feed. Some species in the Firmicutes phylum can produce volatile fatty acids to provide energy for animals. Previous studies have shown that Bacteroidetes and Firmicutes are dominant bacterial phyla in the rumen of ruminant animals such as cattle and sheep [10], consistent with the results of this experiment. In addition, Prevotellaceae and Spirochaetaceae are the dominant bacterial families at the taxonomic level. Members of the Prevotellaceae family are primarily responsible for the degradation of cellulose and hemicellulose in the rumen microbiota. These bacteria can utilize cellulose and hemicellulose in plant cell walls to break them down into smaller sugar molecules, providing substrates for subsequent fermentation processes [31]. Members of the spirochete family mainly participate in the degradation of organic matter and energy recovery in the rumen microbiota. These bacteria have a spiral morphology and can efficiently decompose organic matter in anaerobic environments. In this experiment, the Prevotellaceae family of oat grass decreased with time, indicating that the degradation of cellulose and hemicellulose in oat grass may have been efficiently carried out by Prevotellaceae in the initial stage. Over time, these available substrates gradually decreased, leading to a decrease in the relative abundance of Prevotellaceae. This indicates that the degradable components in oat grass may be rapidly utilized by the rumen microbial community. Prevotella under the phylum Firmicutes and Treponema under the phylum Treponema are dominant bacterial genera in the rumen microbiota, and their activities have a significant impact on the digestive efficiency, nutrient absorption, and overall health status of ruminants [32]. In this experiment, the dominant genera at the genus level were Prevotella and Spirogyra, which is similar to the research results in Liu’s study [9]. In this experiment, the Prevotella genus in oat grass was 44.94% after 4 h. Over time, the relative abundance of Prevotella genus attached to the surface of oat grass feed decreased continuously from 4 to 72 h. This trend is consistent with previous observations on changes in the genus during rumen digestion [33].

Correlation analysis is important for further exploring the degradation of oat grass in the rumen. In three experiments, we demonstrated that microorganisms influence rumen fermentation and animal performance through synergistic interactions [34]. The interactions within microbial communities are crucial for maintaining ecological balance and functional functioning. In this experiment, complex positive and negative correlations were observed between bacterial genera, which may be influenced by various factors including resource competition, metabolite exchange, etc. In addition, a positive correlation indicates that there may be symbiotic relationships between some bacterial genera, which may promote growth or collaborate to complete certain metabolic functions. Negative correlation suggests that there may be competitive relationships between these bacterial genera, which may affect each other’s growth and metabolic activities through resource competition or the influence of metabolic products. The positive correlation between Christensenellaceae and the genera Rikenella and F082 in this experiment may indicate their interdependence in survival and reproduction, possibly because they complement each other in metabolic processes or jointly promote the circulation of certain key nutrients, consistent with the results of Liu et al. [9]. In addition, studies have shown that Rikenella, as a probiotic, has been widely used in probiotic preparations [35]. Christensenellaceae may be associated with a probiotic-rich state, which is related to some beneficial microbial metabolic characteristics and immune responses, helping to maintain the stability of the rumen microbiota. Succinivibrio, Bacillus subtilis, and Prevotella are closely related to the digestion of dietary fiber, among which Succinivibrio and Bacillus subtilis are closely associated in the joint activation and biohydrogenation processes [33]. Prevotella and Rikenella, among other carbohydrate-degrading bacteria, are abundant in the rumen microbiota and can rapidly degrade degradable dry matter and crude protein in roughage [36]. The negative correlation between Sucinivibrio and Christensennellaceae, F082 genera in this experiment, and the negative correlation between Cytomonas and Saccharofermentans and Prevotella may indicate that they compete for the same resources or that their metabolites inhibit each other’s growth. This competitive relationship may lead to changes in the relative abundance of certain species in the community, thereby affecting the functionality of the entire ecosystem.

The rumen microbiota plays an important role in the digestion of ruminants. Studying the correlation between rumen degradation rate and microbiota can help us understand how microorganisms affect feed degradation and nutrient release [37]. Cellulose is an important fiber-degrading bacterium in the rumen, capable of efficiently decomposing cellulose and hemicellulose in plant cell walls. Succinivibrio plays a role in maintaining the acid–base balance of the rumen environment and stabilizing the microbial community. Ruminococcus is involved in the metabolism of starch and sucrose, Saccharofermentans plays a role in degrading metabolites such as C5 branched diacids, and Pseudobutyrivibrio bacteria are associated with propionic acid metabolism in the rumen, affecting the balance of short-chain fatty acids and energy metabolism in the rumen. In the correlation analysis between rumen degradation rate and rumen microbiota, the nutrient degradation of oat grass is positively correlated with Treponema, Ruminococcus, and Cellulose, especially the degradation of DM and CP, which are not only related to the aforementioned bacterial genera, but also to several other bacterial genera such as Prevotella, Saccharofermentas, and Ruminobacter. This may reflect a more complex microbial interaction network, where multiple bacterial genera jointly participate in and promote the degradation of oat grass nutrients. In addition, the positive correlation between ADF degradation and Succinivibrio and Saccharofermentans suggests that these bacterial genera play a role in degrading complex lignin and cellulose structures [38]. The positive correlation between NDF degradation and Succinivibrio further emphasizes the importance of Succinivibrio in degrading recalcitrant plant components [39].

Carbohydrates are one of the carbon sources for rumen bacteria, especially Bacteroidetes and Firmicutes, which play an important role in breaking down complex carbohydrates in the rumen with the help of digestive enzymes [40]. The positive correlation between carbohydrate metabolism pathways and rumen microbiota in this experiment suggests that these bacterial genera may play a key role in specific carbohydrate metabolism pathways, such as the positive correlation between Butyrivbrio and Prevotella with galactose metabolism and the interconversion metabolism pathways of pentose and glucuronide, the positive correlation between Ruminococcus and starch and sucrose metabolism pathways, and the positive correlation between Sucinivibrio and ascorbic acid and glucuronic acid metabolism and C5 branched diacid metabolism, indicating that these bacterial genera play important roles in carbohydrate degradation and energy harvesting [41]. For negative correlations such as the negative correlation between cellulomonas and C5 branch diacid metabolism and propionic acid metabolism, the negative correlation between Saccharofermentas and C5 branch diacid metabolism, and the negative correlation between Treponema and ascorbic acid and aldehyde metabolism, this may indicate that these bacterial genera have opposite roles in these metabolic pathways to those expected, or that they may be more active in other metabolic processes, thereby affecting the activity of these specific pathways [42]. The positive correlation between Butyrivbrio and multiple metabolic pathways is particularly significant, indicating that it may have broad adaptability and roles in the metabolism of various sugars [43].

## 5. Conclusions

This study revealed the dynamic process of interaction between rumen microorganisms and the feed matrix, which provides a theoretical basis for analyzing the mechanism of fiber degradation in ruminants, and has important reference value for optimizing ruminant feed formulation and improving the efficiency of resource utilization.

## Figures and Tables

**Table 1:** Composition and nutrient levels of basal diets (% dry matter basis).

Items	Content (%)	Nutrient Composition ^2^	Content (%)
Leymus chinensis	55	Dry matter	88.69
Corn	31	Crude protein	10.94
Soybean meal	12.2	Neutral detergent fiber	48.66
NaCl	0.5	Acid detergent fiber	24.83
Limestone powder	0.1	Ether extract	2.36
Calcium bicarbonate	0.2	Calcium	0.62
Premix ^1^	1	Phosphorus	0.35
Total	100	Net energy for lactation (MJ/kg DM)	11.67

^1^ Premix (% DM): Fe: 90 mg/kg, Cu: 12.5 mg/kg, Mn: 60 mg/kg, Zn: 90 mg/kg, Se: 0.3 mg/kg, I: 1.0 mg/kg, Co: 0.3 mg, Vitamin A: 15,000 IU/kg, Vitamin D: 5000 IU/kg, Vitamin E: 50 mg/kg. ^2^ The net energy of lactation was calculated value, and others were measured value.

**Table 2:** Degradation rates and degradation parameters of oats DM, CP, NDF, and ADF in the rumen.

Items	DM	CP	NDF	ADF
Ruminal degradation rate/%				
4 h	20.80 ± 4.74 ^e^	24.47 ± 4.91 ^e^	13.37 ± 4.04 ^e^	9.30 ± 2.00 ^e^
8 h	27.60 ± 4.78 ^de^	30.39 ± 5.51 ^de^	19.52 ± 4.15 ^de^	13.04 ± 1.14 ^e^
12 h	34.00 ± 3.93 ^d^	35.75 ± 4.82 ^d^	27.24 ± 4.33 ^d^	18.97 ± 2.53 ^d^
24 h	45.82 ± 3.61 ^c^	48.64 ± 3.32 ^c^	36.93 ± 6.16 ^c^	28.13 ± 2.60 ^c^
36 h	54.72 ± 3.57 ^b^	55.50 ± 4.82 ^bc^	44.88 ± 5.73 ^bc^	34.69 ± 2.05 ^b^
48 h	61.38 ± 4.28 ^ab^	60.56 ± 3.89 ^ab^	50.74 ± 4.65 ^ab^	41.68 ± 2.75 ^a^
72 h	65.76 ± 3.35 ^a^	64.16 ± 3.53 ^a^	55.24 ± 3.92 ^a^	45.32 ± 2.05 ^a^
*p*-value	<0.0031	<0.001	<0.0019	<0.0068
Degradation parameters/%				
“a”	13.12 ± 2.72 ^A^	16.31 ± 3.14 ^A^	6.27 ± 3.29 ^B^	3.33 ± 1.5 ^B^
“b”	57.11 ± 3.13 ^A^	50.7 ± 3.10 ^AB^	52.26 ± 3.55 ^B^	47.42 ± 2.23 ^B^
“c” (%/h)	0.037 ± 0.007	0.042 ± 0.009	0.038 ± 0.009	0.031 ± 0.005
“ED”	47.94 ± 7.23 ^A^	48.69 ± 7.65 ^A^	38.41 ± 8.44 ^AB^	30.24 ± 4.66 ^B^

DM, dry matter; CP, crude protein; NDF, neutral detergent fiber; ADF, acid detergent fiber. “a”, rapidly degraded fraction; “b”, slowly degraded fraction; “c”, degradation rate constant; “ED”, effective degradability. For rumen degradation rates, different lowercase letters (^a–e^) within the same column indicate significant differences (*p* < 0.05). For rumen degradation parameters, different uppercase letters (^A,B^) within the same row indicate significant differences (*p* < 0.05). The same letter or no letter indicates that the difference is not significant (*p* > 0.05).

**Table 3:** Cellulase activity attached to the surface of oat during rumen degradation.

Items	Rumen Retention Time					*p*-Value
	4 h	12 h	24 h	36 h	72 h	
β-GC nmol/min/g	126.72 ± 11.00 ^c^	172.25 ± 18.04 ^b^	159.00 ± 4.69 ^b^	233.50 ± 11.01 ^a^	216.16 ± 3.91 ^a^	<0.001
EG nmol/min/g	186.61 ± 8.09 ^d^	215.96 ± 14.39 ^c^	288.54 ± 10.59 ^a^	284.18 ± 7.29 ^a^	257.63 ± 0.64 ^b^	<0.001
CBH nmol/min/g	281.57 ± 23.71 ^b^	315.83 ± 22.81 ^b^	335.69 ± 15.33 ^a^	325.35 ± 29.36 ^ab^	315.33 ± 28.53 ^ab^	<0.001
NEX nmol/min/g	256.79 ± 32.19 ^d^	451.33 ± 48.33 ^b^	305.04 ± 19.39 ^cd^	339.20 ± 20.71 ^c^	513.76 ± 1.94 ^a^	<0.001

^a–d^ Different lowercase letters in the same row indicate significant differences (*p* < 0.05). β-GC: β-Glucosidase; EG: endo-1,4-β-D-glucanohydrolase; CBH: exo-1,4-β-D-glucanase; NEX: Neutral xylanase.

**Table 4:** Alpha diversity of rumen bacteria attached to oat surfaces.

Items	Rumen Retention Time					*p*-Value
	4 h	12 h	24 h	36 h	72 h	
Chao1	539.31 ± 20.59	484.61 ± 158.5	521.41 ± 44.71	515.51 ± 99.08	424.95 ± 163.54	0.649
ACE	540.17 ± 20.51	482.63 ± 157.85	519.74 ± 43.72	516.05 ± 99.35	423.96 ± 162.57	0.634
Shannon	7.07 ± 0.24	7.15 ± 0.65	7.26 ± 0.40	7.32 ± 0.41	7.12 ± 0.63	0.944
Simpson	0.98 ± 0.01	0.98 ± 0.01	0.98 ± 0.01	0.99 ± 0.01	0.98 ± 0.01	0.573
Goods_coverage	0.9996 ± 0.0001	0.9997 ± 0.0002	0.9996 ± 0.0001	0.9997 ± 0.0001	0.9998 ± 0.0001	0.415

## Data Availability

The data are contained in the article.
